# Basic Research in Bacterial Pathogenesis: “What’s Your Job about, Exactly?”

**DOI:** 10.1371/journal.ppat.1005612

**Published:** 2016-10-13

**Authors:** Isabelle Derré

**Affiliations:** Department of Microbiology, Immunology, and Cancer Biology, University of Virginia School of Medicine, Charlottesville, Virginia, United States of America; The Fox Chase Cancer Center, UNITED STATES

My journey in basic research started off in France, where I received a PhD in microbiology from the Pasteur Institute in Paris. Like many European students, I went overseas for a postdoctoral training and spent four years at Tufts University in Boston, Massachusetts. My original plan was to go back to France, but life happened, and after ten years at Yale University, I recently relocated to the University of Virginia, where I am very excited to start my own laboratory. When I look back at the past 15 years of my scientific career, I see some ups and downs. To be honest, there were probably more downs, and without a genuine passion for what I do and the strong support and guidance from various mentors and colleagues, I would probably not be writing this piece. But most importantly, the ups have carried me through the storms of grants and paper submissions. They are so gratifying and uplifting that no matter how many downs will come my way, I know I have chosen the right career path.

I have been asked many times: “What’s your job about, exactly?” To my colleagues, I can be very specific, and my answer can get down to the molecular level. But when the question comes from nonscientists, that’s another story. Some of my friends joke that I put on a white lab coat and fool around all day with colorful solutions, pipets, and cylinders. While I do have fun in the lab, that’s not how I would describe my professional activities. I am a bacteriologist, which means that I study microorganisms known as bacteria, and I am particularly interested in the ones that are pathogenic to humans. In the pipeline dedicated to the fight against infectious diseases, my job is upstream of patient care and drug development. I focus on understanding the strategies employed by pathogenic bacteria to infect and damage people.

I study the obligate intracellular bacteria *Chlamydia trachomatis*. It is the leading cause of sexually transmitted infection of bacterial origin in the developed world. In women, *Chlamydia* infections are often asymptomatic, and if left untreated, sequelae range from damage of the fallopian tubes, long term pelvic and abdominal pain, ectopic pregnancy, and infertility. Diagnostic tests are available and antibiotics can clear the infection, but reinfection occurs, and in the absence of a vaccine, *Chlamydia* infections remain a public health concern.

Through years of coevolution with humans, *Chlamydia* has learned to survive and replicate in the cytosol of mammalian cells. The bacteria alternate between two physiologically and morphologically distinct forms that dictate whether the bacteria are infectious or in a replicative state. It does so in a membrane-bound compartment, called the inclusion, which protects the bacteria from being detected by the host. The flip side is that the inclusion membrane separates the bacteria from essential nutrients present in the cytosol. To circumvent this, *Chlamydia* possesses a syringe-like apparatus, known as a type III secretion system, which allows the bacteria to inject effector molecules across the inclusion membrane in order to manipulate cytosolic cellular organelles and signaling pathways. These are just a few highlights of the *Chlamydia* developmental cycle.

I decided to study *Chlamydia* because I found its developmental cycle fascinating and I wanted to understand the molecular mechanisms behind it. Why? Partly for the sake of knowledge. Unfortunately, in today’s society, investing in knowledge may not be perceived as one of the highest priorities. In my opinion, however, it is critical to continue our scientific investigations aiming to understand the world around us and “within” us, in the case of intracellular bacteria. Our curiosity-driven research allows for the exploration of new and different ideas, and by following up on discoveries, wherever they lead, basic research scientists contribute to the realization of a never-ending puzzle. Results gathered from basic research will contribute to tomorrow’s textbooks, will advance the knowledge of the next generations, and will hopefully incite them to pursue our endeavor. In addition to contributing to knowledge, I also chose to study *Chlamydia* because of its relevance to human health. Through basic research, the identification of bacterial and host factors supporting pathogen infection constitutes a major avenue for designing novel approaches to therapeutically interfere with the infection process. Therefore, novel therapeutic interventions explored through translational research critically rely on basic research.

Only time will tell whether my research program will significantly contribute to the genesis of knowledge as well as novel therapeutic interventions. We have performed genetic screens to identify host factors that are required for *Chlamydia* intracellular replication. These studies led to the identification of a novel mechanism by which *Chlamydia* potentially hijack host cellular lipids that are important for the completion of its developmental cycle. Furthermore, we have identified a *Chlamydia* type III secretion effector protein that mediates the hijacking process. As we further characterize and understand this pathway, we envision novel approaches for interfering with *Chlamydia* development and therefore blocking the infection process. In addition, because the use of secretion systems is a common theme among some intracellular bacteria, our research program has the potential to shed light onto conserved mechanisms used by other bacterial pathogens. Finally, everything we learn about *Chlamydia* interaction with the host may also help understand basic cellular processes, which in turn may be beneficial to our understanding of noninfectious human diseases. So, although there may not be any immediate applications, we shall not forget that over time, collective knowledge gathered through basic research is the foundation of any kind of translational research and that at the end of day, every little piece of the puzzle matters.

**Image 1:**
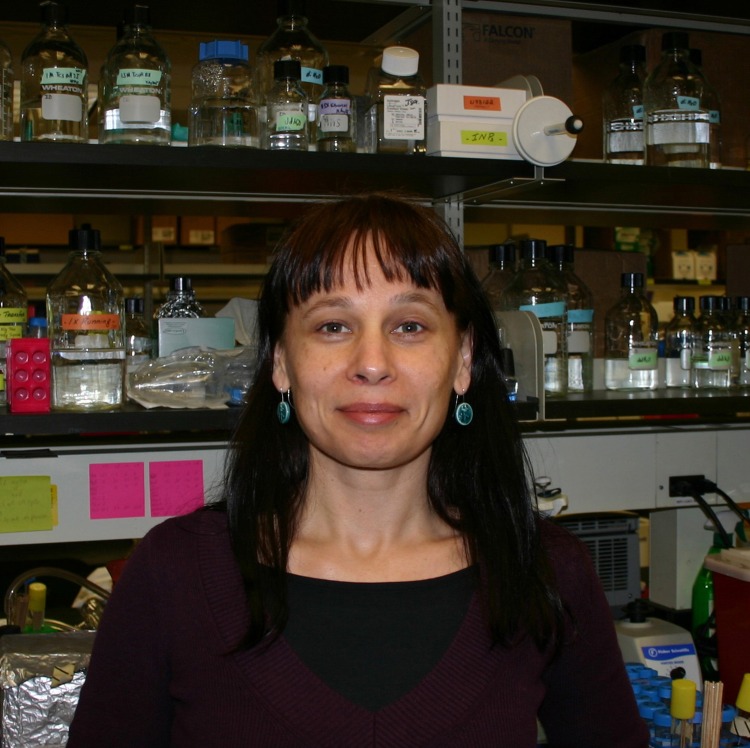
Isabelle Derré.

